# Sex-specific lymphatic responses to estrogen shape atherosclerosis in high-risk mice

**DOI:** 10.3389/fcvm.2026.1699372

**Published:** 2026-03-11

**Authors:** Mona Mesples, Élizabeth Lacroix, Nolwenn Tessier, Maya Farhat, Andreea Milasan, Sara Babran, Cristina Fernandez, Tally Latendresse, Justin Benoit, Valérie Long, Julie Guillemette, Sami El Khakani, Azadeh Alikashani, Charles-Alexandre Leblanc, Marie-Ève Higgins, Vanessa Durocher-Granger, Céline Fiset, Catherine Martel

**Affiliations:** 1 Department of Medicine, Faculty of Medicine, Université de Montréal, Montreal, QC, Canada; 2 Montreal Heart Institute Research Center, Montreal, QC, Canada; 3 Faculty of Pharmacy, Université de Montréal, Montreal, QC, Canada

**Keywords:** atherosclerosis, cardiovascular risk, lymphatic function, estrogen, sex-based differences, menopause

## Abstract

**Introduction:**

Atherosclerosis, the cholesterol-driven inflammatory process underlying cardiovascular disease (CVD), remains the leading cause of death in high-income countries despite major advances in risk factor management. This underscores the urgent need for therapies that directly target plaque development and progression. Recent evidence has uncovered an important role for lymphatic vessels in cardiovascular health: by facilitating reverse cholesterol transport, lymphatics help clear excess cholesterol from arterial walls and influence atherosclerosis from its earliest stages to advanced disease. Enhancing lymphatic pumping before atherogenesis limits plaque formation, while restoring lymphatic function in established atherosclerosis reduces lesion size and promotes stabilization. CVD risk rises sharply after menopause, and lymphedema studies suggest that women experience a more pronounced age-related decline in lymphatic pumping than men, pointing to a potential link with hormonal fluctuations. Hormonal changes throughout life—whether due to aging, therapeutic interventions, or personal choice—are key determinants of CVD vulnerability. Yet, how these changes affect lymphatic transport in individuals predisposed to CVD remains unexplored.

**Methods:**

In this study, age-matched *Ldlr^−/−^* males and ovariectomized females— in which estrogen levels were reduced to mimic the decline observed during menopause— were treated with 17*β*-estradiol (E2) to assess the impact of hormone therapy on *in vivo* lymphatic function and atherosclerosis.

**Results:**

In males, E2 reduced lesion burden and improved lymphatic transport without increasing the expression of key lymphatic endothelial and muscle cell genes involved in vessel integrity and function. In females, estrogen receptor *α*—but not estrogen receptor *β*—was critical for lymphatic vessel function, and its downregulation reduced Flt4 mRNA abundance, a gene essential for lymphatic growth and pumping. E2 impaired lymphatic function in ovariectomized females; however, enhancing lymphatic transport beforehand prevented this effect and reduced atherosclerotic plaque formation.

**Conclusion:**

Our findings reveal that estrogens modulate lymphatic function and atherosclerosis differently according to sex and baseline hormonal status. These results suggest that lymphatic function may contribute to the interplay between hormonal changes and cardiovascular risk, supporting the development of more targeted therapeutic strategies for populations undergoing hormonal transitions.

## 1 Introduction

Sex-based differences in the incidence of cardiovascular diseases (CVD) have been increasingly documented over the past decades. While women tend to develop CVD later in life than men, their cardiovascular risk increases markedly 10 years following menopause (1). This physiological condition is associated with a significant drop in the levels of estrogen, a hormone that is thought to be responsible for women being protected from CVD prior to menopause (2, 3).

Atherosclerosis is the predominant cause of CVD. Characterized by cholesterol accumulation and an exacerbated inflammatory response within the arterial walls, it remains prevalent despite advanced therapies (4, 5). Martel et al. previously highlighted the prerequisite role of the lymphatic vessels in the removal of excess cholesterol from the artery wall during reverse cholesterol transport (6). Milasan et al. subsequently reported that dysfunction of collecting lymphatic vessels occurs prior to the onset of atherosclerosis in low-density lipoprotein receptor-deficient (*Ldlr^−/−^*) mice (7). *Ldlr^−/−^* mice are a well-established model of severe hypercholesterolemia, characterized by advanced plaque formation under high-fat diet (8, 9). Moreover, we have showed that LDLR deficiency *per se* in lymphatic endothelial cells (LECs) alters the expression of key factors required for LEC integrity, suggesting a direct mechanistic link between LDLR signaling and lymphatic dysfunction (10). Our team also demonstrated that early stimulation of the lymphatic pumping in *Ldlr^−/−^* mice with vascular endothelial growth factor-C [VEGF-C (152s)], a selective VEGF receptor-3 (VEGFR-3) agonist, before the administration of a pro-atherosclerotic regimen, improved long-term lymphatic vessel capacities while limiting plaque progression and contributing to its stabilization thereafter (11).

Extensive research on lymphatic transport focuses mainly on a disease called lymphedema, which is characterized by limb fluid and fat accumulation (12). Primary lymphedema disproportionately affects women (12). Women also demonstrate a more pronounced age-related decline in lymphatic pumping pressure compared to men, indicating a potential causal link with hormonal fluctuations (13). In the context of secondary lymphedema post–breast cancer, Morfoisse et al. used a mouse model of unilateral secondary lymphedema and reported that estrogens are critical for re-establishing lymphatic endothelial cell growth following tissue repair (14). They reported that this beneficial effect is mediated by an activation of the estrogen receptor *α* (ER*α*) and is antagonized by tamoxifen, a selective estrogen receptor modulator (SERM) used in first line for women who develop breast cancer.

Fluctuations in estrogen are not solely restricted to women experiencing menopause. Hormonal changes can arise naturally or be induced by medications or surgical interventions, either for therapeutic purposes or for gender transition (15). Gender-affirming hormone therapy (GAHT) is intended to align physical appearance with self-perceived gender, ultimately aiming to reduce gender dysphoria and improve psychological well-being (16). Estrogen-based therapies are currently used across multiple clinical contexts, including the management of menopausal vasomotor symptoms (VMS) (17), the treatment of conditions such as prostate cancer (18) and osteoporosis (19), and as a component of GAHT (20). The interplay between menopausal hormone therapy (MHT) and CVD has been widely debated. Results from clinicals trials such as the Heart and Estrogen/progestin Replacement Study (HERS) (21) and Women's Health Initiative (WHI) (22) suggest a negative effect of MHT on the cardiovascular health of post-menopausal women. Subsequent investigations, however, outweigh these conclusions by acknowledging the importance of both the timing of therapy initiation and the type of hormone administrated on cardiovascular outcomes (23, 24). While our understanding of the impact of MHT on CVD continues to grow, the influence of GAHT on cardiovascular health across the lifespan remains understudied, despite emerging evidence that trans women may be at high risk of CVD (25).

The recent recognition of the lymphatic system as an important contributor to CVD highlights a critical gap in our knowledge: how hormonal changes influence lymphatic transport in individuals already at risk of CVD remains largely unexplored. Considering the interplay between lymphatic function and hormonal variations, and the well-documented association between low estrogen levels and increased CVD risk, we hypothesize that lymphatic dysfunction may represent a key link between estrogen decline and atherogenesis. This study examines sex-specific differences in the effects of 17*β*-estradiol (E2) on lymphatic function and atherosclerotic burden in a high-risk, proatherogenic mouse model, with the ultimate goal of identifying strategies to limit atherosclerosis-associated damage in a sex- and hormone-dependent manner (7, 10). We observed that while E2 treatment had beneficial effects on rescuing lymphatic function, and limiting atherosclerosis and hypercholesterolemia in male mice, it worsened lymphatic function in age-matched ovariectomized female mice, most likely in an ER*α*-dependent mechanism affecting the VEGFR-3 signaling pathway. However, increasing lymphatic function prior to ovariectomy prevented the decay in lymphatic pumping observed in E2-treated mice and limited the development of atherosclerotic lesions.

## 2 Material and methods

### 2.1 Mice

This study was conducted in *Ldlr^−/−^*, *Erα^−/−^*, *Erβ^−/−^*, and wild-type (WT) mice, all originally obtained from the Jackson Laboratory. All strains were on a C57BL/6 background. Animals of both sexes were bred and housed in a specific pathogen-free environment at the Montreal Heart Institute animal facility under a 12-hour light/dark cycle, with unrestricted access to water and food. Mice were maintained on a standard rodent diet (Teklad Global 19% Protein Extruded, Inotiv, 2019S; 19.2% protein, 9% fat, 44.9% carbohydrates) and switched to a high-fat diet (HFD; adjusted-calorie diet containing 0.2% total cholesterol and 42% fat, Harlan, 88,137) according to the experimental protocol. All mice were housed in groups of 2–4 animals in individually ventilated cages within a positive-pressure room maintained at 20  °C with 40%–60% relative humidity. Each cage contained wood chip bedding to keep animals dry and prevent direct contact with the cage floor. Environmental enrichment was provided in all cages, including a small house, a ring, and nesting material (Enviro Dry®, tissue paper, and Alpha Twist). Cages were changed under a laminar flow hood every two weeks for cages housing 1–2 mice and weekly for cages with three or more mice. The *Ldlr^−/−^* model was chosen because it is widely used to study atherosclerosis, as these mice develop hypercholesterolemia and atherosclerotic lesions when fed a high-fat diet. In addition, our previous study has shown that LDL receptor deficiency itself can impair lymphatic function early on (10), making this model particularly relevant for investigating the interplay between lipid metabolism and lymphatic physiology. *Erα^−/−^* and *Erβ^−/−^* mice were included to assess the specific contributions of estrogen receptor signaling to lymphatic and vascular function, given the established roles of ER*α* and ER*β* in cardiovascular physiology and sex hormone-mediated regulation of vascular homeostasis. WT mice were also used to evaluate the impact of reduced ER*α* expression specifically in endothelial cells. All experiments were performed in accordance with the Canadian Council on Animal Care guidelines and approved by the Montreal Heart Institute (MHI) Animal Care Committee (protocols #2020-14-01 and 2021-14-02).

### 2.2 Experimental design

Seven-week-old female *Ldlr^−/−^* mice were randomly assigned to experimental groups and treated to enhance lymphatic function prior to ovariectomy. Mice received intraperitoneal (IP) injections of VEGF-C (152s) at a dose of 50 ng per 25 g of body weight [purified recombinant rat VEGF-C protein (152s), Fitzgerald, catalog no. 30R-AV006-22] or vehicle control (phosphate-buffered saline, PBS), administered three times per week for four consecutive weeks. At 11 weeks of age, female mice underwent either bilateral ovariectomy (OVX) or sham surgery (same anesthetic regimen, aseptic prep, and positioning as OVX, with the same dorsal skin and muscle incisions at both sites), according to the standardized ovariectomy procedure at the MHI (26, 27). *Ldlr^−/−^* male mice received the same sham procedure. Concomitantly, all mice were switched from a standard chow diet to a high-fat diet (HFD—adjusted calories diet, 0.2% total cholesterol and 42% fat, Harlan, 88137) until sacrifice to promote development of atherosclerotic lesions. One week later, all mice were implanted with 17*β*-estradiol, the most predominant estrogen (Innovative Research of America, SE-121), or placebo (Innovative Research of America, SC-111) by making a 0.5 cm caudo-lateral skin incision in the neck, into which the implant was inserted subcutaneously. The implant delivered 0.1 mg of estradiol, corresponding to 80 µg/kg per day, over 60 days, to mimic of circulating estradiol in young female mice (28–32). Mice were sacrificed at 20–21 weeks of age.

In another set of experiments, C57BL/6 female mice lacking either estrogen receptor *α* (*Erα^−/−^)* or estrogen receptor *β* (*Erβ^−/−^*) were used, with age- and sex-matched WT mice as controls. These mice were kept on a standard chow diet until sacrifice at 12 weeks of age. Finally, to assess whether the effects observed were attributable to *Esr1* (e.g., gene coding for ER*α*) deletion on endothelial cells, a targeted knockdown was used. Ten-week-old female C57BL/6 WT mice received 250 µL IP injections of 1 × 10^11^ of adeno-associated virus type 1 (AAV1) encoding either a short hairpin RNA (shRNA) against *Esr1* (shEsr1) or a scramble control (shCtrl; based on Addgene #85741). AAV1 constructs were produced as previously described (10) and sequences are listed in Table 1. Mice remained on standard chow diet and were sacrificed at 12 weeks of age.

**Table 1:** Sequences used for AAV1-shRNA and qPCR primers.

shRNA	
Gene target	Sequences (5’ – 3’)
shEsr1	GCTCCTGTTTGCTCCTAACTT
shCtrl	GTTCAGATGTGCGGCGAGT
qPCR primers	
Gene target	Sequences (5’ – 3’)
*Actb*	Forward: GATGTATGAAGGCTTTGGTC
	Reverse: TGTGCACTTTTATTGGTCTC
*Lyve1*	Forward: CCACAACTCATCCGACACCT
	Reverse: TCTGTTGCGGGTGTTTGAGT
*Prox1*	Forward: GACGTGAAGTTCAACAGATG
	Reverse: TTGTTGTAGTGCATGTTGAG
*Flt4*	Forward: AGCTCTACATATCACCGAAG
	Reverse: CACAGTTGTAATATCTGGCTG
*Vegfd*	Forward: CCCCAGAAGAAGATGAATGTCC
	Reverse: CACAGAGAGTGGGTTCCTGG
*Cdh5*	Forward: TCATCAAACCCACGAAGTCCC
	Reverse: GGGTCTGTGGCCTCAATGTA
*Pdpn*	Forward: AGATAAGAAAGATGGCTTGC
	Reverse: AACAACAATGAAGATCCCTC
*Esr1*	Forward: GCTGAACCGCCCATGATCTA
	Reverse: TTCAAGTCCCCAAAGCCTGG
*Acta2*	Forward: CCTCTGGACGTACAACTGGTATT
	Reverse: GCCCTCATAGATAGGCACGTTG

AAV1, adeno-associated virus type 1; shEsr1, short hairpin RNA targeting estrogen receptor *α*; shCtrl, scramble short hairpin RNA used as control; *Actb*, gene coding for *β*-actin; *Lyve1*, gene coding for lymphatic vessel endothelial hyaluronan receptor 1; *Prox1*, gene coding for prospero homeobox protein 1; *Flt4*, gene coding for vascular endothelial growth factor receptor 3; *Vegfd*, gene coding for vascular endothelial growth factor D; *Cdh5*, gene coding for VE-Cadherin; *Pdpn*, gene coding for podoplanin; *Esr1*, gene coding for estrogen receptor 1/estrogen receptor alpha; *Acta2*, gene coding for actin alpha 2.

Lymphatic function was assessed on mice anesthetized with 2.0% or 5.0% isoflurane in 0.5 L/min O_2_. Then, mice were euthanized under isoflurane either by cardiac puncture or CO_2_ inhalation depending on the experiment performed, followed by perfusion with 15 mL PBS. Heart, liver, aorta, uterus, ovaries, lymphatic vessels, lymph nodes, and visceral adipose tissue (e.g., epididymal and periovarian white adipose tissues harvested from the abdominal cavity) were subsequently harvested and stored as previously described for further analysis (6, 7, 10, 11, 33, 34). Organs and visceral adipose tissue were weighed on a precision scale and reported relative to body weight for each animal. Outcome assessment and data analysis were blindly performed to reduce subjective bias.

### 2.3 Assessment of lymphatic function

#### 2.3.1 *In vivo* measurement of lymphatic vessel contraction

Lymphatic vessel contractions were measured as previously described (10, 11, 34). An incision was made around the skin of the upper hind limb of anesthetized mice to allow the skin to be delicately retracted, exposing the underlying muscle. Two intradermal injections (10 μL each) of ovalbumin-Alexa Fluor 647 (2 mg/mL, Fisher, O34781) were administered on each side of the footpad, and the paw was manually pumped three times to facilitate the movement of the fluorescent ovalbumin into lymphatic vessels. Warm saline solution was applied to the paw to keep it hydrated throughout the imaging. After a 10-minute waiting period to allow stabilization of the lymphatic contractions, images were acquired using an Axiozoom V.16 microscope (Zeiss). A total of 326 images were acquired with 0.8 s exposure over a 10 min recording period. Images were stabilized using the Template Matching ImageJ™ plugin and three to five regions of interest were drawn along the lymphatic vessels using LymphPulse 3.0, a Matlab™-based software used to quantify contractions. Fluorescence intensity (in arbitrary units, AU), from which the background was subtracted, was plotted and displayed peaks and valleys corresponding to lymphatic contractions. Contraction frequency was calculated using the Matlab algorithm: (Np + Nv)/(2*dt), where *Np* and *Nv* respectively represent the number of peaks and valleys, and *dt* is the analysis time.

#### 2.3.2 FITC painting assay

Immune cell transport through the lymphatic network was evaluated via the assessment of the capacity of dendritic cells to migrate from the periphery to draining lymph nodes (LNs), as described previously (7, 11, 33). An immune response was instigated by applying a solution on top of shaved skin, containing fluorescein isothiocyanate (FITC), dibutyl phthalate and acetone. Mice were euthanized 18 h later, and corresponding skin-draining LNs were harvested and enzymatically digested in collagenase D for 25 min at 37 °C. Cells were filtered in a 70 µm cell strainer, washed, counted and stained for analysis by flow cytometry with a BD FACSCelesta (BD Biosciences). Conjugated antibodies CD11b PerCp-Cy5.5 (BioLegend, 101228), CD11c BV786 (BD Bioscience, 563735) and MHCII PE (Tonbo Biosciences, 50-5321-U025) were used. The number of FITC-positive dendritic cells that migrated to the corresponding draining LN and the percentage of FITC-positive cells in the draining LN were analyzed with FlowJo™ software (Tree Star Inc.) (Supplementary Figure S1).

#### 2.3.3 Lymphatic vessel leakage

Lymphatic vessel leakage was assessed following the injection of 10 µL of Evans blue (EB) dye in the dermis of each side of the footpad of anesthetized mice, as previously described (7, 11, 33). Manual paw pumping was performed to promote the uptake of the dye by the popliteal collecting lymphatic vessels. The skin over the hind limb was carefully removed following an incision, allowing the exposure of the lymphatic vessels, which were imaged using a Stemi 508 microscope (Zeiss). Dye leakage was depicted as either present or absent.

### 2.4 Quantitative RT-PCR

Liver, ovaries and lymphatic vessels were harvested and snap-frozen in liquid nitrogen for qPCR analysis. To retrieve popliteal and flank lymphatic vessels, an intradermal injection of EB dye was performed in the paw of the mice, as described above. Collecting lymphatic were harvested, cleaned *ex vivo*, snap-frozen in liquid nitrogen, and stored at −80 °C until further use. Livers, ovaries and lymphatic vessels were ground in liquid nitrogen and homogenized in Trizol. Multiple lymphatic vessels from four mice per group were pooled to ensure sufficient RNA yield. RNA was extracted according to the manufacturer's protocol (Invitrogen, 12183025) and stored at −80 °C. Reverse transcription was carried out using the High-Capacity cDNA Reverse Transcription Kit (ThermoFisher Scientific, 4368814). The QuantStudio™ 3 (ThermoFisher Scientific) was used to perform quantitative PCR with 10 ng of complementary DNA and Itaq™ Universal SYBR® Green Supermix (Bio-Rad, 1725121). Amplification conditions were as follow: 94 °C for 10 s and 60 °C for 45 s, for 40 cycles. Relative gene expression was calculated using the 2^−*ΔΔ*CT^ method, normalized to the housekeeping gene *Actb*. The sequences of the primers used in this study are shown in Table 1.

### 2.5 Quantitative analysis of plasma cholesterol and lipoprotein fractions

Blood was collected with ethylenediaminetetraacetic acid (EDTA) by cardiac puncture. Plasma was recovered by centrifugation at 2,400 g for 10 min. Lipoproteins were separated at 4 °C by size exclusion chromatography on a Superose 6 10/300 GL column (GE Healthcare) connected to an AKTA Explorer Fast Protein Liquid Chromatography (FPLC) system. The column was equilibrated with PBS (Wisent, 311-010-CL). For each mouse, plasma was loaded onto the column using a 100 µL sample loop and separated at a flow rate of 0.1 mL/min. Approximately 65 fractions of 0.3 mL were collected and cholesterol was measured (Wako, 439-17501) in the first 60 fractions, according to the manufacturer's protocol.

### 2.6 Atherosclerotic lesion quantification

Aortas and hearts were harvested and fixed overnight in 4% paraformaldehyde at 4 °C. Aortas were transferred to PBS, then cleaned *ex vivo* to remove excess surrounding fat and longitudinally cut open along the outer curvature. Hearts were placed in PBS containing 30% sucrose (w/v) for two days at 4 °C and then the buffer was replaced with PBS alone. The hearts subsequently were immersed in optimal cutting temperature compound (OCT) and stored at −80 °C. Serial 8 µm-thick cryosections were made of the aortic sinuses. *En face* aortas and aortic sinuses were stained with Oil-red-O (ORO) (Sigma, O-0625) to assess neutral lipid deposition and identify necrotic regions, defined as ORO-negative areas within the atherosclerotic lesions. Images were captured with a Stemi 508 microscope (Zeiss) and lesions were quantified using the software ZEISS ZEN 3.7.

### 2.7 Statistics

Experiments are presented as mean ± standard error of the mean (SEM), with *n* indicating the number of mice, unless otherwise specified. Statistical analyses were conducted using PRISM Version 10.2.3 software (GraphPad Software Inc). The Shapiro–Wilk test was used to assess normality. *p*-values were calculated using either parametric (one- or two-tailed Student T tests for comparisons between two groups, or one-way ANOVA using multiple comparison correction as appropriate for multiple groups) or non-parametric (Mann–Whitney test for comparisons between two groups, with results presented as median and min/max) tests. For contingency table analyses, Fisher's exact test was used. Statistical significance was defined as *p* ≤ 0.05.

## 3 Results

### 3.1 E2 treatment improved lymphatic function through non-genomic mechanisms in *Ldlr*^−/−^ male mice

**Figure 1:**
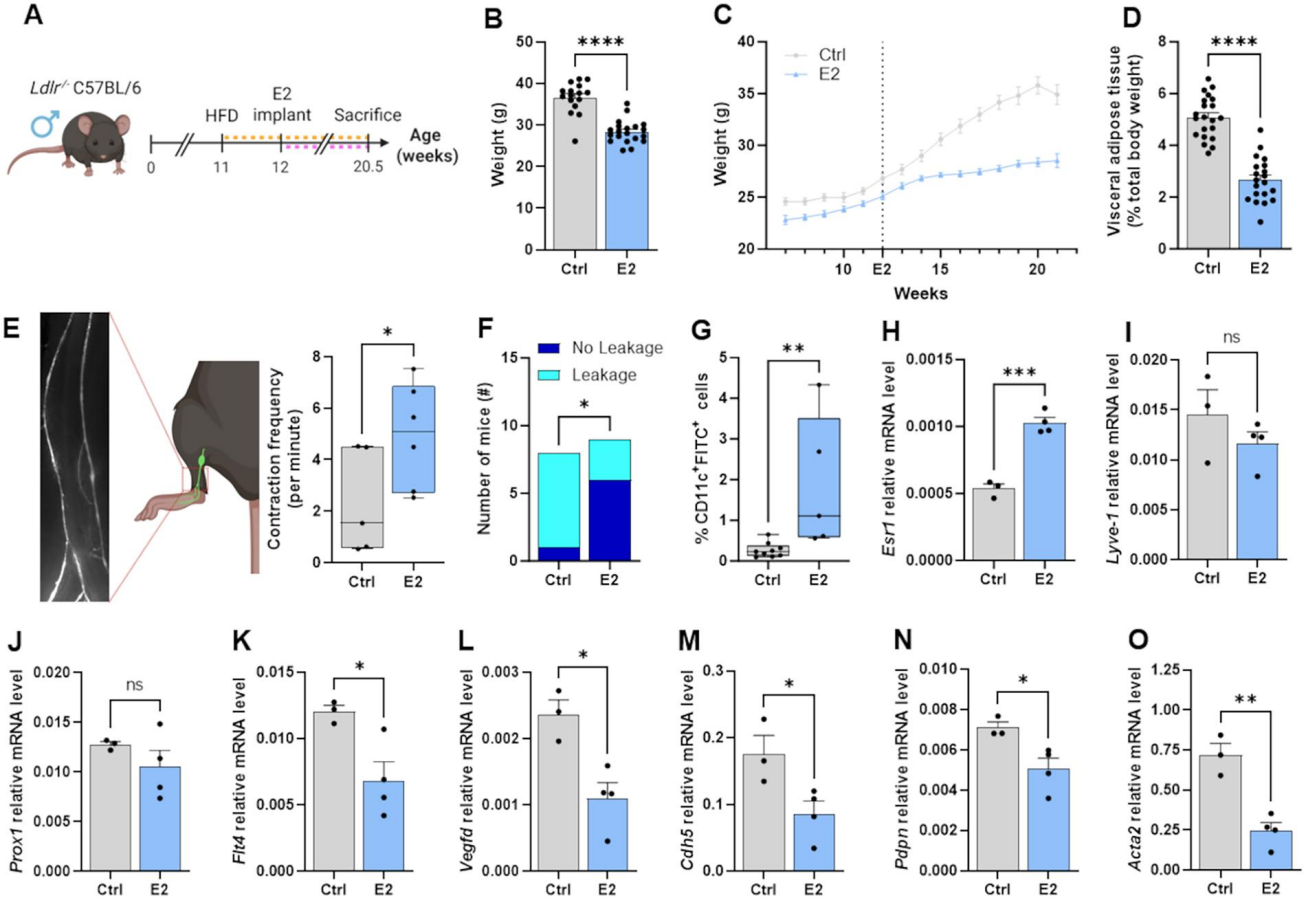
Estradiol supplementation improved systemic lymphatic function while down-regulating the abundance of LEC and LMC mRNA of key regulatory proteins in atherosclerotic Ldlr-/- male mice. **(A)** Experimental design. Ldlr-/- male mice were fed a high-fat diet (HFD) starting at 11 weeks of age. Estrogen (E2) supplementation began one week later. Lymphatic function was assessed in mice immediately prior to sacrifice at 20.5 weeks, after which organs were harvested. Orange and pink dashed lines represent HFD and E2 implant durations, respectively. **(B)** Total body weight at the time of sacrifice, **(C)** over 21 weeks and **(D)** percentage of visceral adipose tissue on the total body weight were measured. Popliteal collecting lymphatic vessel **(E)** contraction frequency, **(F)** leakage and **(G)**. dendritic cell migration from the skin to the draining lymph node were reported to assess lymphatic function. Transcripts for **(H)**. estrogen receptor-α (Esr1) and **(I–O)**. lymphatic genes were quantified in harvested collecting lymphatic vessels. Data points represent individual animals; qPCR points = pools of 4 mice (2–4 vessels). ns = *p* > 0.05, *p < 0.05, **p < 0.01, ****p < 0.0001. Esr1, estrogen receptor α; Lyve1, lymphatic vessel endothelial hyaluronan receptor 1; Prox1, prospero homeobox 1; Flt4, vascular endothelial growth factor receptor 3; Vegfd, vascular endothelial growth factor D; Cdh5, VE-Cadherin, Pdpn: Podoplanin, Acta2: α-2-actin.

Estrogen use in men is increasing, primarily through GAHT in transgender women and as treatment for medical conditions, with important implications for cardiovascular health (25). To identify the impact of hormonal changes on lymphatic transport in individuals already at risk of CVD, we first sought to determine the effect of E2 supplementation in atherosclerotic *Ldlr^−/−^* male mice. In male mice (Figure 1A), estrogen supplementation during high fat feeding efficiently reduced body weight gain and the percentage of visceral adipose tissue (Figures 1B–D). It also improved collecting lymphatic vessel contractility (Figure 1E) and reduced their leakiness (Figure 1F). In line with these observations, the transport of immune cells through lymphatic vessels was improved by E2 treatment, as demonstrated by the higher proportion of double-positive CD11c^+^ FITC^+^ cells in the lymph nodes draining the skin site where the FITC solution was applied (Figure 1G, Supplementary Figure S1). Quantification of ER*α* (e.g., *Esr1*) mRNA confirmed the expected upregulation in mice supplemented with E2 (Figure 1H). The abundance of transcripts involved in lymphangiogenesis and lymphatic cell differentiation like *Lyve1* (lymphatic vessel endothelial hyaluronan receptor 1) and *Prox1* (prospero homeobox 1) was not impacted by E2 treatment (Figures 1I,J) in collecting lymphatic vessels. However, *Flt4*, coding for VEGFR-3, and *Vegfd* coding for one of its ligands, VEGF-D, were significantly less abundant in E2-treated male mice (Figures 1K,L). Transcripts for the adherent cell junction protein VE-Cadherin (*Cdh5*) and lymphatic endothelial cell marker podoplanin (*Pdpn*) were also less abundant than in control-treated male mice (Figures 1M,N). Collecting lymphatic vessels are characterized by lymphatic muscle cell (LMC) coverage, and we have observed that the transcript for smooth muscle *α*-2 actin (*Acta2*), a protein important for LMC contractility, was less abundant in the E2-treated group (Figure 1O). Based on these observations, E2 supplementation in atherosclerotic *Ldlr^−/−^* male mice enhanced lymphatic vessel function at the systemic level, while unchanging or even downregulating transcripts for proteins involved in lymphatic vessel growth and function. This suggests that ER*α* activation could enhance lymphatic transport through non-genomic or indirect mechanisms in atherosclerotic male mice.

### 3.2 Estrogen treatment reduced the atherosclerosis burden in *Ldlr*^−/−^ male mice

**Figure 2:**
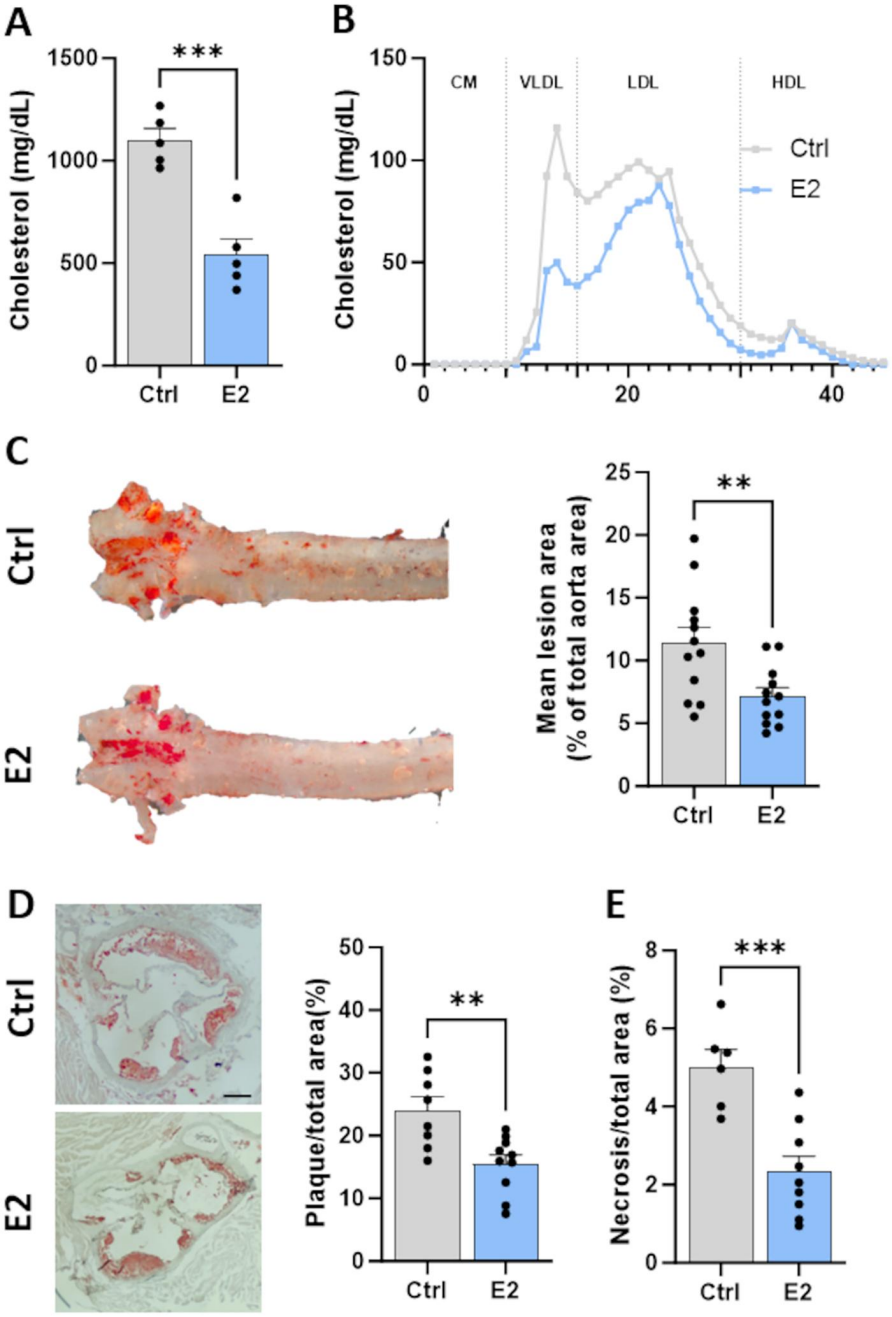
Effect of estradiol supplementation during high-fat diet feeding on cholesterol levels and plaque accumulation. **(A)** Plasma total cholesterol and **(B)** lipoprotein profile were measured. **(C)** Representative images of en face oil-red-O (ORO) staining of the aorta and quantification of atherosclerotic plaque burden, expressed as the percentage of total aortic area. **(D)** Representative images of aortic cross sections stained with ORO and quantification of atherosclerotic plaque burden and **(E)**. necrotic core area, expressed as percentage of total sinus area and as percentage of total plaque area, respectively. Data points represent individual animals. Scale bar represents 200 μm. **p < 0.01, ***p < 0.001. CM, chylomicron; VLDL, very-low density lipoprotein; LDL, low-density lipoprotein; HDL: high-density lipoprotein.

Given that boosting lymphatic function prior to atherogenesis sustains lymphatic pumping and restrains plaque formation in *Ldlr^−/−^* high fat fed mice (11), we hypothesized that improvements in lymphatic function induced by E2 would, in turn, limit plaque formation. Thus, we next assessed the subsequent impact of E2 treatment on the atherosclerosis burden in atherosclerotic male mice. E2 diminished plasma total cholesterol levels by 50% (Figure 2A). Size exclusion chromatography revealed that this decrease was associated with a reduction in very-low density lipoprotein (VLDL) and low-density lipoprotein (LDL) levels (Figure 2B). Quantification of neutral lipids in atheromatous lesions showed that E2 reduced plaque accumulation in both the whole aorta (Figure 2C) and in the aortic sinus (Figure 2D). In addition, mice treated with E2 had on average half the plaque necrosis observed in control-treated male mice (Figure 2E). These results highlight the positive impact of estrogen therapy through reduction of atheromatous lesions in atherosclerotic male mice.

### 3.3 ER*α*, but not ER*β*, is critical for lymphatic vessel function in female mice

**Figure 3:**
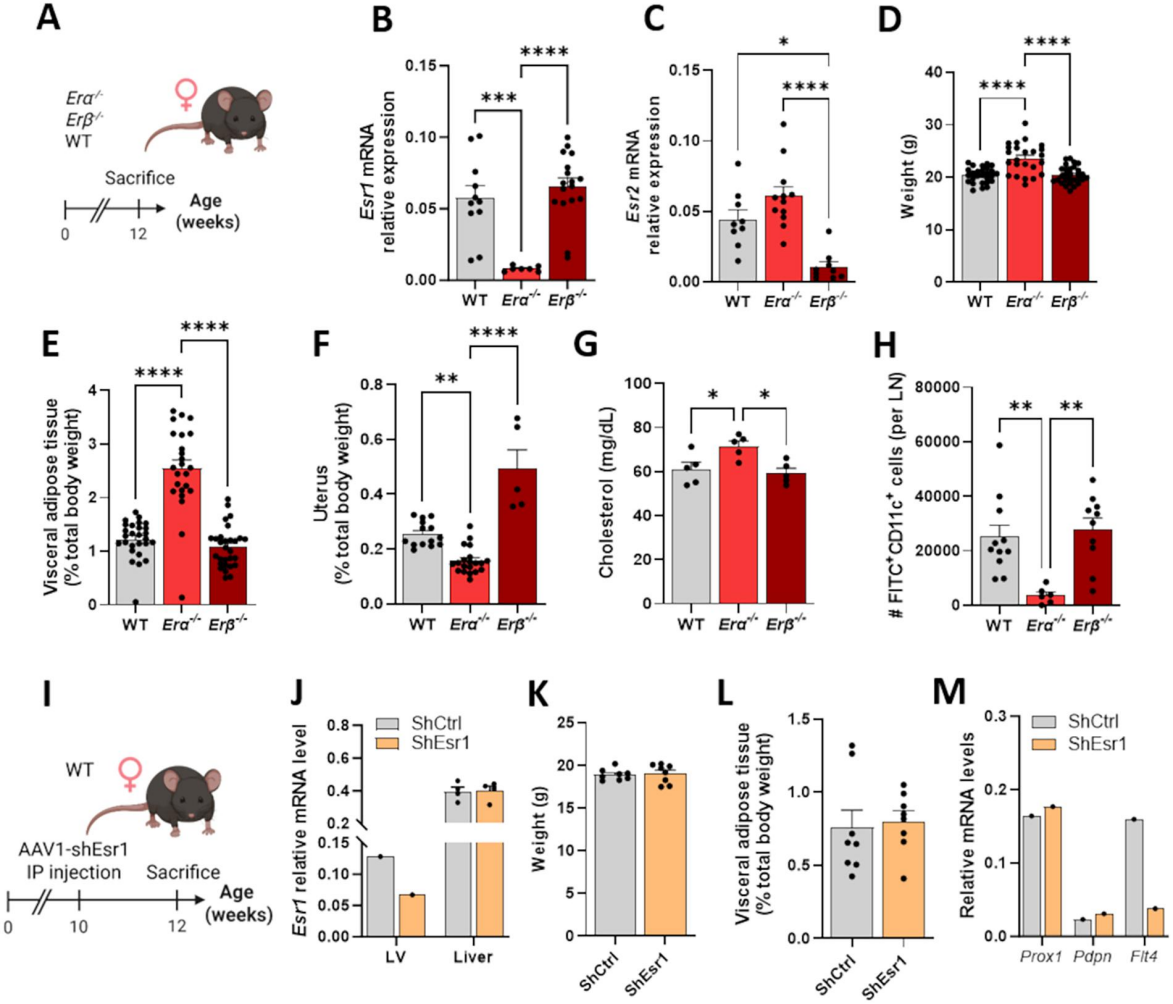
Deficiency in ER*α*, but not ER*β*, led to impaired lymphatic function and down-regulation of VEGFR-3 in female mice. **(A)** Experimental design. *Erα^−/−^* and Er*β*^−/−^ female mice were kept on a standard chow diet until sacrifice at 12 weeks, after which organs were harvested. **(B,C)** Measurement of *Esr1* and *Esr2* by qPCR in ovaries confirmed the ER deficiency in both models, respectively. **(D)** Total body weight was assessed. Weight of **(E)** the visceral adipose tissue and **(F)** the uterus, each expressed as percentage of total body weight. **(G)** Cholesterol plasma levels were assessed. **(H)** Dendritic cell migration from the skin to the draining lymph node was measured to assess systemic lymphatic function. **(I)** Experimental design for adeno-associated virus type 1 (AAV1) encoding a short hairpin RNA (shRNA). Wild-type (WT) female mice, fed on a standard chow diet, received IP injections of AAV1 encoding either a shRNA against Esr1 (shEsr1) or a scramble control (shCtrl) at 10 weeks of age. Organs were harvested after sacrifice at 12 weeks. **(J)** qPCR confirmed the targeted depletion of *Esr1*. LV were pooled from 4 mice (2–4 vessels). **(K)** Total body weight was measured and **(L)**. weight of visceral adipose tissue was expressed as percentage of total body weight. **(M)** Transcripts of lymphatic genes were quantified in harvested lymphatic vessels (pool of 4 mice). Data points represent individual animals; LV qPCR points = pools of 4 mice (2–4 vessels). *p < 0.05, **p < 0.01, ***p < 0.001, ****p < 0.0001. LV, lymphatic vessel; ER, estrogen receptor; Esr1, estrogen receptor α; Esr2, Estrogen receptor β; Prox1, prospero homeobox 1; Flt4, vascular endothelial growth factor receptor 3; Pdpn, Podoplanin.

Whereas estradiol acts through both ER*α* and ER*β*, their beneficial effects on both blood and lymphatic endothelial cells in female mice appears to be mediated through ER*α* (14, 35–37). To further understand receptor-specific contributions to lymphatic function *per se*, we used ER*α*– and ER*β*–deficient female mice with wild-type (WT) controls maintained on a chow diet for twelve weeks (Figure 3A). Knockout efficiency was confirmed by reduced *Esr1* or *Esr2* (ER*α* and ER*β*, respectively) mRNA abundance in ovaries (Figures 3B,C). Morphological parameters further supported model integrity (38, 39), as *Erα^−/−^* mice displayed increased body weight and adiposity, reduced uterine size, and more elevated plasma cholesterol than WT mice, while *Erβ^−/−^* mice showed no significant changes (Figures 3D–G). Functional analysis of lymphatic transport revealed a clear divergence between the two models: whereas *Erβ^−/−^* mice exhibited preserved lymphatic function similar to WT animals, *Erα^−/−^* mice showed a significantly reduced migration of FITC-positive dendritic cells from the skin to the draining lymph node compared to both *Erβ^−/−^* and WT mice (Figure 3H, Supplementary Figure S1). These findings establish that lymphatic dysfunction is specifically driven by ER*α* deletion, underscoring a protective role of ER*α*, but not ER*β*, in maintaining lymphatic transport in female mice.

Because these knockout models reflected systemic deletion of ERs, we acknowledge that any lymphatic changes observed could be influenced by whole-body effects. To specifically address the role of ER*α* in endothelial cells, we next generated a targeted approach using an AAV1 encoding shEsr1, designed to selectively suppress ER*α* expression in endothelial cells (Figure 3I). The selectivity of this strategy was confirmed by showing that the abundance of *Esr1* mRNA was reduced in harvested lymphatic vessels but not in the liver (Figure 3J). Unlike the systemic model, AAV1-mediated knockdown did not alter body weight or visceral fat accumulation (Figures 3K,L), supporting its tissue specificity. At the molecular level, the abundance of *Prox1* and *Pdpn* mRNA remained unchanged, whereas ER*α* knockdown led to a threefold decrease in *Flt4* mRNA, which encodes VEGFR-3 (Figure 3M). Although these exploratory results should be confirmed in further studies, this downregulation likely underlies the lymphatic dysfunction observed in absence of ER*α*, as VEGFR-3 is critical for lymphatic growth and pumping. Together, these findings indicate that ER*α* signaling within the lymphatic vasculature is essential for maintaining optimal lymphatic function in female mice.

### 3.4 Pre-treatment with VEGF-C protected lymphatic function and reduced the atherosclerotic plaque in E2-supplemented ovariectomized mice

Post-menopausal women are at high risk of CVD, and the use of MHT remains a concern for many women. Using an ovariectomized mouse model of estrogen depletion, widely used to study postmenopausal hormonal changes (40), we aimed to investigate how estrogen influences lymphatic transport in atherosclerotic mice, and whether enhancing lymphatic function prior to ovariectomy can modulate lymphatic function and atherosclerotic plaque formation following an MHT regimen.

**Figure 4:**
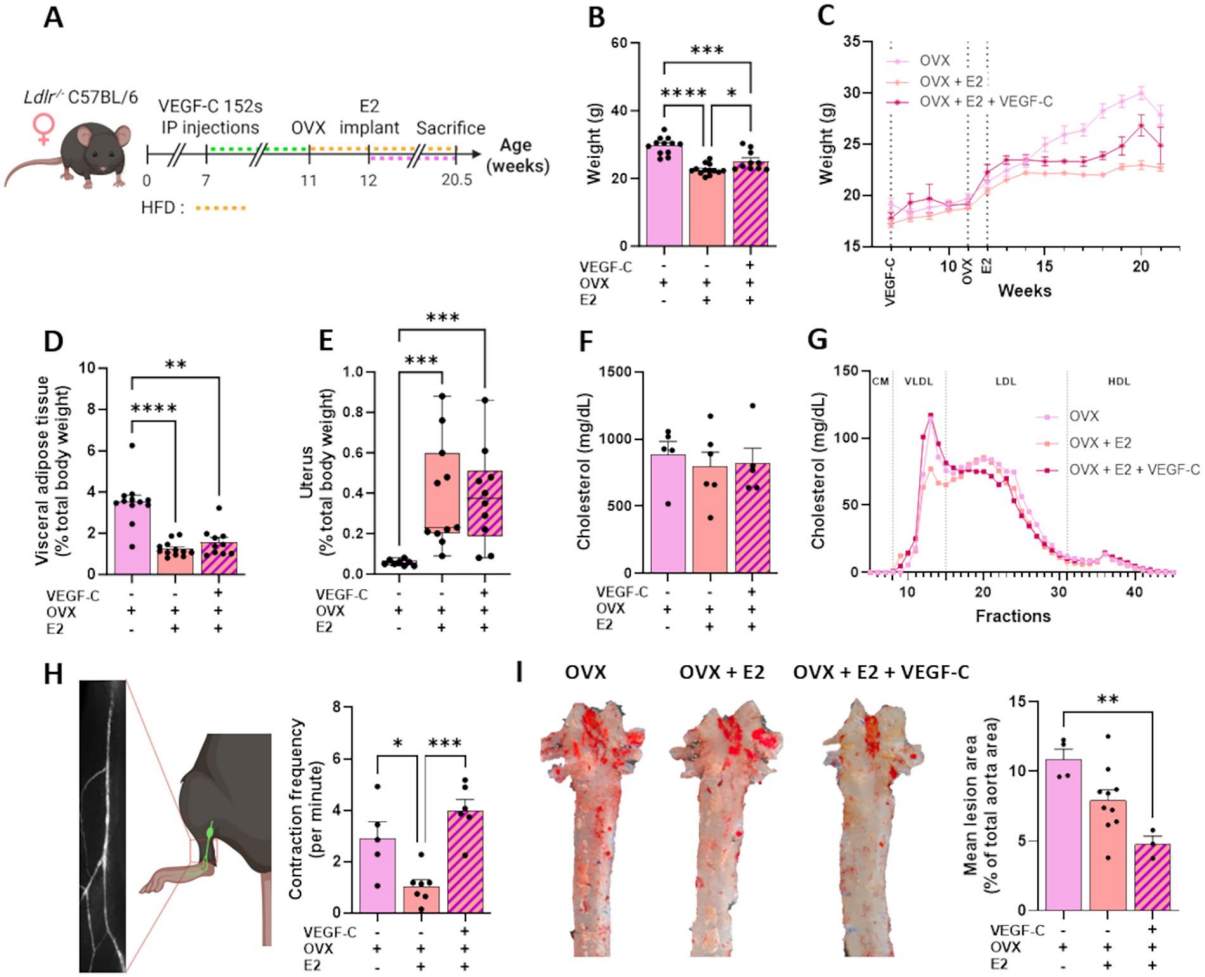
VEGF-C 152s treatment prior to ovariectomy limited the negative effects of subsequent E2 supplementation on lymphatic function and plaque burden in *ldlr^−/−^* mice. **(A)** Experimental design. 7 weeks old *Ldlr^−/−^* female mice received IP injections of VEGF-C 152s (e.g., VEGF-C) or vehicle. At 11 weeks, mice underwent either bilateral ovariectomy (OVX) or sham surgery, and switched on a high-fat diet (HFD). Estrogen (E2) supplementation began one week later. Lymphatic function was assessed in mice immediately prior to sacrifice at 20.5 weeks, after which organs were harvested. Green, orange and pink dashed lines represent VEGF-C 152s injections, HFD, and E2 implant durations, respectively. **(B)** Total body weight at the time of sacrifice and **(C)** over 21 weeks were reported. **(D)** Weight of visceral adipose tissue and **(E)** weight of the uterus, each expressed as percentage of total body weight. **(F)** Plasma total cholesterol and, **(G)** lipoprotein profile were assessed. **(H)** Contraction frequency of the popliteal collecting lymphatic vessels was measured by intravital microscopy. **(I)** Representative images of *en face* oil-red-O staining of the aorta and quantification of atherosclerotic plaque burden, expressed as percentage of total aortic area. Data points represent individual animals. *p < 0.05, **p < 0.01, ***p < 0.001, ****p < 0.0001. HFD, high-fat diet; OVX, ovariectomy; VEGF-C, vascular endothelial growth factor C; CM, chylomicron; VLDL, very-low density lipoprotein; LDL, low-density lipoprotein; HDL, high-density lipoprotein.

We investigated early VEGF-C 152s-mediated lymphatic stimulation in ovariectomized *Ldlr^−/−^* mice, with or without estradiol treatment to model menopause and MHT (Figure 4A). As expected, estradiol reduced body weight gain and visceral adiposity in ovariectomized mice, while preserving uterine weight. These effects were generally maintained with a VEGF-C 152s pre-treatment (Figures 4B–E). Total cholesterol remained unchanged across treatments (Figure 4F), except for a modest reduction in VLDL cholesterol with estradiol, not observed when VEGF-C 152s is given prior to ovariectomy (Figure 4G). Strikingly, estradiol markedly decreased lymphatic contraction frequency in ovariectomized mice, indicating impaired lymphatic function in this post-menopausal, proatherogenic model (Figure 4H). Pre-treatment with VEGF-C 152s fully prevented this dysfunction. Consistently, E2 alone had minimal impact on aortic plaque accumulation, whereas VEGF-C 152s injections prior to E2 implantation significantly reduced lesion development (Figure 4I). Overall, these results demonstrate that MHT can impair lymphatic function, and that early lymphatic stimulation effectively prevents this dysfunction and attenuates atherosclerotic progression in post-menopausal female mice.

## 4 Discussion

Throughout life, hormonal fluctuations play a key role in shaping CVD susceptibility (18, 24, 25, 41–46). However, the underlying causes, the time of onset and progression, and the mechanisms involved remain poorly characterized. There is a growing need to clarify how and when context-dependent hormone therapies modulate atherosclerosis and the subsequent cardiovascular outcomes through aging (47, 48). In this study, we provide insights on the role of estrogen on lymphatic function, a crucial player in atheroprotection, of high-CVD risk mice. Our findings reveal that E2 treatment improves lymphatic transport through non-genomic mechanisms in atherosclerotic *Ldlr^−/−^* male mice while concomitantly reducing the plaque burden. However, in female mice, downregulation of *Flt4* mRNA, a key driver of lymphatic growth and pumping, occurs upon loss of ER*α*, but not ER*β*, highlighting the critical role of ER*α* in lymphatic vessel function in female mice. Importantly, we show that while E2 impairs lymphatic function in *Ldlr^−/−^* ovariectomized mice, boosting lymphatic transport before the ovariectomy preserves contractions during E2 treatment and limits atherosclerotic plaque.

E2 has been reported to provide a protective effect against atherosclerosis, largely through the very first step in the disease onset, namely endothelial dysfunction (37, 49, 50). In blood vessels, E2 binds to both ER*α* and ER*β*, with ER*α* playing a predominant role in mediating its protective effects in endothelial cells, thereby contributing to its atheroprotective action in *Ldlr^−/−^* mice (51). The ER*α*-estrogen binding acts on endothelial function by initiating various signaling pathways, including those involving protein kinases and intracellular calcium (35–37). These pathways influence cell function through fast non-genomic mechanisms and transcriptional regulation. Few years ago, Morfoisse et al. published that ER*α*, but not ER*β*, are present in lymphatic endothelial cells (14). On the lymphatic endothelium, both nuclear and membrane ER*α* activities were shown to alter signaling and transcription. In blood vessels, ER*α* activation is known to regulate several genes, including *LDLR* (35), which we have identified as a key player in lymphatic endothelial cell membrane lipid changes and subsequent lymphatic vessel contractility (10). E2 has also been shown to increase the abundance of transcripts for lymphatic markers such as *Flt4*, *Vegfd* and *Lyve1*, and, in turn, to enhance lymphangiogenesis (14). Similarly, we herein report that, in female WT mice, lymphatic dysfunction is promoted by knocking out ER*α*, but not ER*β*. Our models of ER depletion confirmed the metabolic effect of ER*α*, as described by the increased body and adipose tissue weight of mice lacking this receptor (38). The systemic and the targeted depletion of *Esr1* were mirrored by the impairment of lymphatic function and induced a reduction of *Flt4* expression in collecting lymphatic vessels that requires further confirmation.

In atherosclerotic *Ldlr^−/−^* male mice, our findings stand in contrast to these observations. We report that E2 reduced the expression of lymphatic endothelial and muscle cell function key genes and had no effect on lymphatic lineage markers, despite the expected increase in *Esr1* in lymphatic vessels. The impact of estrogen on male body weight and visceral fat was consistent with the decrease reported in the literature (47, 48, 52). As systemic lymphatic transport and pumping capacity are, however, enhanced by the E2 treatment in our atherosclerotic mouse model, the beneficial effects on lymphatic transport could be mainly due to non-genomic signaling pathways. Estradiol activates rapid, non-transcriptional ER*α* pathways that leads to the stimulation of endothelial NO synthase (eNOS) (53, 54). Of interest, NO is a radical messenger that is essential to control lymphatic pumping (55–57). We hypothesize that NO might be implicated in the variations of lymphatic vessel contractibility observed under E2 treatment and should be further investigated.

In the context of secondary lymphedema post-breast cancer, Morfoisse et al. reported that estrogens are critical for re-establishing lymphatic endothelial cell growth following tissue repair, and they concluded that secondary lymphedema is worsened by SERM therapy. In our study, E2 supplementation in *Ldlr^−/−^* ovariectomized mice clearly reduces lymphatic vessel contractile capacity. Although E2 has been shown to have protective effects on lymphatic vessel leakage (14), we show here that E2 can worsen lymphatic transport in *Ldlr^−/−^* mice, which are already known to have lymphatic dysfunction even before the onset of atherogenesis (7). Collectively, as the main lymphatic defect observed in atherosclerotic *Ldlr^−/−^* mice resides in its impaired contractility, these data suggest that the impact of E2 on lymphatic function may differ depending on the basal E2 status and whether dysfunction primarily occurs in initial or collecting vessels.

Over the past decades, the relationship between MHT and CVD risk has become better understood. North American medical societies now recommend MHT, in appropriately selected patients, primarily for the treatment of VMS (e.g., hot flashes, night sweats, heart palpitations) as well as for the prevention of bone loss and genitourinary symptoms (17, 58, 59). The decision to initiate therapy should consider factors such as age, time since menopause onset, and baseline CVD risk. Nevertheless, VMS affect approximately 75% of women during the menopausal transition and persist up to 10 years (60, 61). It is therefore essential to elucidate the precise mechanisms by which MHT may adversely affect women at high risk of CVD, and to identify strategies to limit these potential harmful effects. Early lymphatic stimulation with VEGF-C 152s injection improves lymphatic function and later limits plaque progression in this *Ldlr^−/−^* mice (11). Given the prevalence of lymphatic dysfunction in women, we aimed to assess the pertinence of preventive stimulation of the lymphatic system before the onset of menopause and the initiation of hormonal therapy. We observed that boosting lymphatic function prevents the adverse effect observed with the E2 treatment. This finding highlight, for the first time, the importance of implementing preventive strategies targeting the lymphatic network prior to the initiation of MHT in high-risk individuals.

E2 supplementation reduced lesion size and necrosis in atherosclerotic male mice, consistent with the beneficial effects of E2 therapy on atherosclerosis in *Ldlr^−/−^* mice (62). Moreover, reduction in total plasma cholesterol, as well as VLDL and LDL cholesterol levels observed following E2 treatment, are in line with previously reported lipid profile alterations in trans women (63). In female mice, an early lymphatic stimulation prior to ovariectomy and estradiol implant reduced lesion size by 50%, boosting the effect of estrogen alone. As VEGF-C 152s increases systemic lymphatic function, lipid absorption by the lacteals is also improved (11), increasing VLDL levels in the plasma. Similar to E2 (51), the atheroprotective effects associated with enhanced lymphatic function appear to occur independently of plasma cholesterol levels. Importantly, boosting lymphatic function prior to ovariectomy counteracts the detrimental effects of E2, highlighting lymphatic transport as a potential therapeutic target before menopause.

Taken together, our results highlight the complexity of how E2 therapy may differentially influence the lymphatic system and cardiovascular health, acting as friend or foe, depending on biological sex, genetic predisposition to CVD, and environmental factors. Thus, therapeutic strategies involving estrogens must be tailored to hormonal status. Hence, women who are candidates for hormone replacement therapy may first benefit from interventions aimed at boosting lymphatic function, thereby preventing lymphatic dysfunction–associated complications such as chronic inflammatory diseases like atherosclerosis. These findings pave the way for more effective and tailored strategies to mitigate CVD risk during hormonal changes throughout life.

## 5 Limitations

Atherosclerosis is a complex, multifactorial disease involving interconnected processes such as lipoprotein metabolism, reverse cholesterol transport, and inflammation. While murine models provide valuable insights, they cannot fully replicate all aspects of human atherosclerosis, particularly in terms of plaque instability (64). These limitations should be considered when extrapolating findings to human pathology. Moreover, the effects of sex hormones in mice have been shown to vary according to factors such as genetic background (65). Consequently, the interpretations drawn from this model should be viewed in light of these variables and validated through additional studies.

## Data Availability

The original contributions presented in the study are included in the article/Supplementary Material, further inquiries can be directed to the corresponding author.

## Supplementary material

Supplementary Figure S1 Flow cytometry gating strategy and representative data for dendritic cell migration assessment. The population of interest was identified by double-positive CD11c+ FITC+ events.
