# Supplementary figures and images for: Sex-specific lymphatic responses to estrogen shape atherosclerosis in high-risk mice
Source: Front Cardiovasc Med. 2026 Mar 11;13:1699372. doi: 10.3389/fcvm.2026.1699372 (PMC13012992; doi:10.3389/fcvm.2026.1699372)

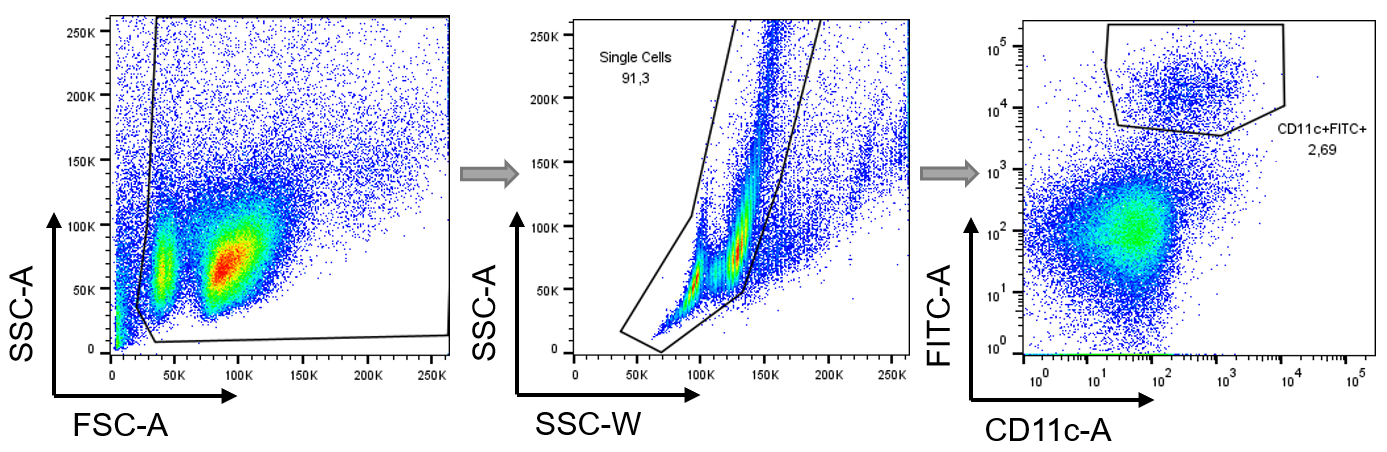

Supplement: Supplementary Figure S1 — Flow cytometry gating strategy and representative data for dendritic cell migration assessment. The population of interest was identified by double-positive CD11c+ FITC+ events. [file Image1.tif]
